# Research on the Strength Properties and Microscopic Mechanism of Loess Stabilized by the Combined Use of MICP Technology and Plant Straw

**DOI:** 10.3390/ma18050992

**Published:** 2025-02-24

**Authors:** Kun Wang, Haoshuang Niu

**Affiliations:** 1 College of Geological Engineering and Geomatics, Chang’an University, Xi’an 710061, China; kunwang@chd.edu.cn; 2 School of Civil Engineering, Henan Polytechnic University, Jiaozuo 454000, China; 3 Key Laboratory of Intelligent Construction and Safety Operation and Maintenance of Underground Engineering in Henan Province, Jiaozuo 454000, China

**Keywords:** MICP technology, osmotic conditions, unconfined compressive strength, plant straw, loess

## Abstract

There are many drawbacks in traditional loess-strengthening technology. MICP (microbially induced calcium carbonate precipitation) technology provides a new approach to loess management, but there are few studies on loess solidification and a lack of engineering application research and verification. This study investigated the strength and microscopic mechanisms of loess solidified by the application of MICP technology combined with plant straw. The permeability conditions of loess for MICP technology were derived, and multiple sets of experiments were conducted using specific loess, *Bacillus pasteurii*, cementing solution, plant straw, and other materials. The experiments explored shear strength, unconfined compressive strength, microscopic properties, plant growth adaptability, and factors affecting bacterial growth. The results indicated that within the temperature range of 25–35 °C, the concentration and urease activity of *Bacillus pasteurii* were significantly affected by temperature, with the highest bacterial concentration observed at 30 °C. During scaled-up cultivation, increasing the inoculation ratio prevented a significant decrease in the urease activity of individual bacterial strains, and a 1% inoculation ratio was generally sufficient to meet the experimental requirements. When the loess density was 1.7 g/cm^3^ and 1.8 g/cm^3^, the cohesive force and internal friction angle in the experimental groups with added bacterial solution were increased by approximately 30% and 50% and 15% and 5%, respectively, indicating that MICP technology can significantly enhance the shear strength of loess.

## 1. Introduction

Traditional loess consolidation and treatment technologies have been widely applied in practical projects in the Loess Plateau region, playing an important role and achieving good results, but there are also shortcomings, such as high cost, difficulty, complexity of operation, considerable engineering required, ecological damage, and environmental protection [1]. Microbially induced calcium carbonate precipitation (MICP) technology is a novel concept and introduces a new approach to treating and safeguarding loess slopes. By harnessing the mineralization and reinforcement capabilities of microorganisms, this technology promotes the formation of calcium carbonate crystals both within and on the surfaces of adjacent soil particles, effectively binding loose particles together to create a cohesive whole [2]. This improves the overall strength and integrity of a slope and does not produce pollutants in the process of reaction, with energy savings, environmental protection, simple operation, low cost, environmental friendliness, and other characteristics.

Since the birth of MICP technology, scholars from various countries have employed it in their research, and a considerable quantity of data has emerged. At present, the technology is widely used in various fields, with a wide range of application prospects, such as soil reinforcement, dust and sand fixation, cultural relic restoration, foundation reinforcement and improvement, concrete restoration, anti-seepage plugging, and heavy metal pollution prevention and control [3,4]. Regarding soil reinforcement, Zhang Shisan and his team demonstrated that microbial perfusion can effectively solidify aeolian sand and significantly enhance its engineering prospects, with a maximum unconfined compressive strength of 7.13 MPa [5]. Li and colleagues further investigated the impact of varying concentrations of cementing and bacterial fluids on aeolian sand. They discovered that an increase in cementing fluid concentration led to higher calcium carbonate content in the solidified layer, resulting in a lower permeability coefficient and elevated unconfined compressive strength [6]. Yang et al. employed MICP technology to treat aeolian sand and conducted wind tunnel tests. Their findings revealed that this treatment effectively mitigated wind erosion, thereby improving the wind erosion resistance of aeolian sand [7]. Domestic and foreign scholars have achieved fruitful research results with MICP technology, but most of the current research focuses on indoor laboratory conditions, with few practical engineering application studies, and the reliability of practical engineering application of MICP technology lacks field test verification.

Most of the existing studies on soil solidified by MICP technology focus on sandy soil, and there are few studies on solidified loess. The pores of loess are small. After solidification using MICP technology, only a solidified layer of a fixed thickness is able to form on the surface [8,9,10,11], which makes it difficult to achieve deep-level solidified treatment of loess. There are few reports on the practical engineering application of loess reinforcement and its integration with vegetation. There are also potential environmental hazards, such as the production of calcium carbonate and also the formation of other substances, and this greatly reduces the advantages of MICP technology, limiting its scope and application scenarios. Aiming to address these problems, we studied the strength characteristics and microscopic mechanisms of MICP technology and plant straw in the co-curing of loess. The permeability conditions of loess under MICP technology were derived, followed by a series of experiments. After treatment with MICP technology, the pores of the loess samples were filled with noticeable calcium carbonate particles that were more tightly arranged, and the degree of calcium cementation was significantly improved, indicating that MICP technology effectively enhanced the microstructure of the soil. Mercury injection test results showed that the total pore volume and average pore size of the loess treated with MICP decreased and both the permeability and porosity were reduced, suggesting that calcium carbonate crystals effectively filled the pores between soil particles. MICP technology can significantly improve the strength and stability of loess, providing a new method for loess reinforcement.

## 2. Derivation of Loess Permeability Conditions Based on MICP Technology

### 2.1. MICP Technology

MICP, i.e., microbially induced carbonate deposition, is mainly used by strains such as *Bacillus pasteurii*, basophils, amino acid bacteria, etc., and is widely used in liquefiable solidification [12,13]. This paper mainly introduces Bacillus pasteurensis, which can use urease produced by its own metabolism and raise the pH value of the environment by producing ammonia ions, resulting in Ca^2+^ and CO_3_^2−^ interaction to form calcium carbonate precipitation. The primary process involved is outlined as follows [11].

Urease can hydrolyze urea to produce NH_3_ and CO_2_, and chemical reactions such as (1) and (2).

(1) $$\mathrm{Cell}+{\mathrm{Ca}}^{2+}\to \mathrm{Cell}-{\mathrm{Ca}}^{2+}$$

(2) $$\mathrm{CO}{\left({\mathrm{NH}}_{2}\right)}_{2}+H_{2}O\to 2{\mathrm{NH}}_{3}+{\mathrm{CO}}_{2}$$

NH_3_ dissolves in water to produce NH^4+^ and OH^−^, as per chemical Equation (3). It is worth mentioning that the OH^−^ generated in this process increases the pH value and supersaturation of the solution, providing conditions for the precipitation of CaCO_3_ crystals [14,15,16], which is also the focus of most studies at present.

(3) $${\mathrm{NH}}_{3}+H_{2}O\to {{\mathrm{NH}}_{4}}^{+}+{\mathrm{OH}}^{-}$$

CO_2_ reacts with OH^−^ to produce HCO^3−^, and the chemical reaction formula is shown as (4):

(4) $${\mathrm{CO}}_{2}+{\mathrm{OH}}^{-}\to {\mathrm{HCO}}_{3}^{-}$$

When Ca^2+^ is present in the environment, Ca^2+^ will react with the generated HCO^3−^ and OH^−^ to generate CaCO_3_ crystals. The chemical reaction formula is as in (5):

(5) $${\mathrm{Ca}}^{2+}+{\mathrm{HCO}}_{3}^{-}+{\mathrm{OH}}^{-}\to {\mathrm{CaCO}}_{3}↓+H_{2}O$$

Microbially induced carbonate precipitation (MICP) involves the formation of calcium carbonate on soil surfaces or building structures through the interaction of urease—produced by microorganisms during their metabolic processes—with calcium ions and nitrogen-source nutrients present in the environment [17]. Unlike calcium carbonate synthesized through traditional chemical methods, MICP-induced calcium carbonate forms particles of various shapes under the regulation of enzymes and organic molecules secreted by microorganisms. These particles exhibit robust gelling and filling properties, capable of cementing loose particles into a cohesive whole with specific mechanical attributes. Consequently, the pores within the soil, particularly those between soil particles, are filled with calcium carbonate, which reinforces and enhances the weak soil foundation [18,19,20,21,22]. This process ultimately improves the soil’s bearing capacity. In this process, the strength and reaction speed have the advantages of controllability, and the method can avoid the disturbance of the foundation in the construction process and realize in situ reinforcement, which has considerable competitiveness compared with the traditional method.

### 2.2. Derivation of Applicable Conditions for Permeability Parameters

Calcium carbonate precipitation is produced by the reaction of carbonate ions with calcium salt solution after the hydrolysis of urea has been promoted by bacterial solution. Therefore, the whole process is in a liquid state, that is to say, if one wants to produce calcium carbonate in the soil, one first needs to meet a corresponding solution in the grouting process that is able to enter the soil pores, which entails requirements for the permeability of the soil. The permeability of granular media is the result of the combined action of external factors (hydraulic gradient) and internal factors (granular matter itself and fluid). Therefore, the permeability can synthesize the requirements of MCP technology for effective particle size, pore diameter, particle composition, and hydrodynamic parameters, etc. This paper deduces the permeability conditions suitable for MICP technology.

Basic assumptions:

(1) Only the above reactions occur in the process of microbial cement bonding.
(2) Calcium carbonate precipitation does not affect the total volume of the liquid.
(3) Grout liquid is incompressible homogeneous isotropic Newton fluid, ignoring the filtration effect of time-varying grout with viscosity [23,24].

According to the curing process and assumptions (2) and (3), the volume of grout does not change. The volume of bacterial fluid and cementing fluid injected each time is *β* and *α* times of the soil pore volume, respectively. The whole curing process is completed through *N* cycles of grouting, then the total amount of grout (bacterial fluid and cementing fluid) during curing is as follows [17]:

(6) $$V_{\mathrm{UC}}=N(\alpha +\beta )n_{0}V$$

The volume of cementing fluid injected during curing is:

(7) $$V_{C}=N\alpha n_{0}V$$

According to Equation (5), the molar ratio of calcium ions corresponding to the generated calcium carbonate precipitation and calcium salt is 1:1. Calcium carbonate yield *S* is defined as the ratio between the actual calcium carbonate precipitation mass and the theoretical calculated value [25]:

(8) $$S=m_{{\mathrm{CaCO}}_{3}}/C_{{\mathrm{Ca}}^{2+}}N\alpha n_{0}VM_{{\mathrm{CaCO}}_{3}}$$

Define the amount of calcium carbonate precipitation actually produced in a unit of grout soil:

(9) $$m_{{\mathrm{CaCO}}_{3}}^{\prime }=m_{{\mathrm{CaCO}}_{3}}/V$$

According to Formulas (7)–(9), the curing frequency of the step-by-step grouting cycle is determined as:

(10) $$N=\frac{m_{{\mathrm{CaCO}}_{3}}^{\prime }}{\alpha Sn_{0}C_{{\mathrm{Ca}}^{2+}}M_{{\mathrm{CaCO}}_{3}}}$$

Under the premise of assumptions (6) and (7), it can be seen that the volume of grouting liquid does not change (which can be called quantitative grouting), and the slurry flow *Q* conforms to Darcy’s law [26,27]:

(11) Q=qA

The three-dimensional form of seepage velocity *q* is:

(12) q=kJ=−k∇ϕ

After *N* times grouting, the permeability coefficient decreased to:

(13) $$k_{N}=\xi k_{0}$$

Assuming that the soil permeability decreases linearly after each grouting and the change in permeability during each grouting cycle solidification is ignored [28,29,30,31], then the permeability coefficient after each solidification is reduced to:

(14) $$\Delta k=\left(k_{0}-k_{N}\right)/N$$

Then, the permeability coefficient after the *x* grouting is:

(15) $$k_{x}=k_{0}-x\Delta k=k_{0}(N-x+\xi x)/N$$

It can be seen from the above that the slurry capacity of each grout is:

(16) $$V_{\mathrm{UC}}/N=(\alpha +\beta )n_{0}V=Qt_{x}$$

Links (11), (12), (15), (16) are available:

(17) $$t_{x}=\frac{(\alpha +\beta )n_{0}LN}{k_{0}(N-x+\xi x)J}$$

Therefore, the total grouting time including *N* stepwise grouting is:

(18) $$t_{S}=\frac{(\alpha +\beta )n_{0}LN}{k_{0}J}\overset{N}{\underset{x=1}{\sum }}\frac{1}{\left(N-x+\xi x\right)}$$

The permeability conditions that meet MICP are mainly considered from two aspects. One is the sustainability of the pulp. In the initial stage of grouting, the permeability is fast. After several episodes of grouting, the permeability gradually decreases with the formation of calcium carbonate precipitation in the soil, and the grouting time increases each time [32] until the grouting cannot be completed within the time allowed by the activity of the bacterial solution or working conditions [33]. Then, the NTH grouting time must meet:

(19) $$t_{N}⩽[t]$$

By substituting Equation (17) into Equation (19), the permeability condition 1 is:

(20) $$k_{0}⩾\frac{(\alpha +\beta )n_{0}L}{\xi [t]J}$$

Therefore, the required amount of calcium carbonate generated per unit of soil mass can be determined according to the specific curing strength requirements [34]. Then, the mass of calcium carbonate generated in the unit soil mass after *N* times grouting is:

(21) $$m_{{\mathrm{CaCO}}_{3}}^{\prime }⩾{\left[m_{{\mathrm{CaCO}}_{3}}^{\prime }\right]}_{cr}$$

Substituting Equation (10) into permeability condition 2:

(22) $$n_{0}⩾\frac{{\left[{m_{{\mathrm{CaCO}}_{3}}}^{\prime }\right]}_{cr}}{\alpha SC_{{\mathrm{Ca}}^{2}}M_{{\mathrm{CaCO}}_{3}}N}$$

By substituting Equation (22) into Equation (20), the permeability condition 3 is obtained:

(23) $$k_{0}⩾\frac{L}{M_{{\mathrm{CaCO}}_{3}}}\frac{\alpha +\beta }{\alpha JC_{{\mathrm{Ca}}^{2+}}N[t]}\frac{{\left[{m_{{\mathrm{CaCO}}_{3}}}^{\prime }\right]}_{cr}}{\xi S}$$

### 2.3. Analysis of Water Absorption of Loess

In the process of calcium carbonate precipitation induced by microorganisms, there are a lot of carbonates in loess, which may affect the metabolic activities of microorganisms and the precipitation efficiency of calcium carbonate. Excessive carbonate may lead to supersaturation of the reaction environment, thus inhibiting the formation of new calcium carbonate precipitation and affecting the effect of MICP technology.

**Figure 1:**
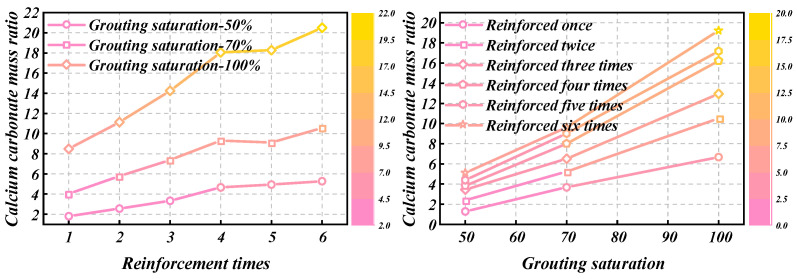
Calcium carbonate mass ratio of loess samples with different grouting saturation.

The relationship between calcium carbonate content of loess samples with different grouting saturation and reinforcement times is shown in Figure 1.

As Figure 1 shows, with the increase in reinforcement times, the calcium carbonate mass ratio of loess samples under each grouting saturation also increased, and the increasing trend of calcium carbonate mass ratio became slow. The calcium carbonate mass ratio of loess samples with 100%, 70%, and 50% grouting saturation increased by 17.7%, 16.7%, and 15.3%, respectively. The increasing ratio showed a decreasing trend with the decrease in grouting saturation.

**Figure 2:**
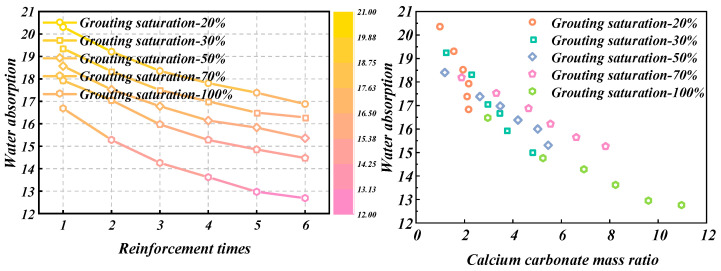
Test results of water absorption of sand samples with different grouting saturation.

The measured relationship curves between water absorption and reinforcement times of sand samples with different grouting saturation are shown in Figure 2.

As shown in Figure 2, with a fixed grouting saturation, the water absorption rate gradually decreases as the number of reinforcement cycles increases, with the most significant effect observed at 100% saturation. For samples reinforced with MICP, microorganisms deposit calcium carbonate in the soil pores through biological processes, filling the pore structure of the soil and thereby reducing the size and porosity of the soil pores, leading to a decrease in the soil’s water absorption rate. When the number of reinforcement cycles is the same, the higher the saturation of the sample, the higher the content of calcium carbonate generated, reaching a maximum of about 11%. Consequently, the lower the porosity, the lower the water absorption rate.

**Figure 3:**
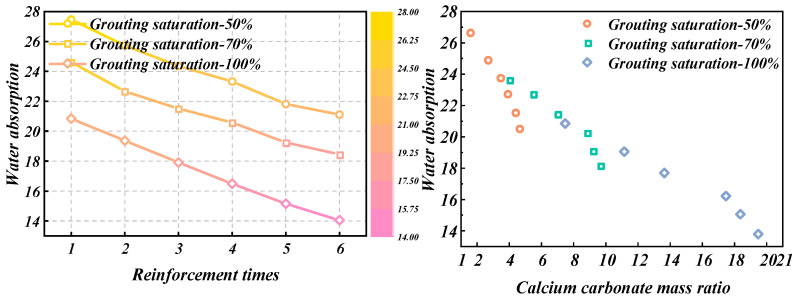
Water absorption test results of loess samples with different grouting saturation.

Figure 3 displays the relationship curves depicting how the water absorption of loess samples varies with reinforcement times, based on different grouting saturation levels.

As shown in Figure 3, with the same grouting saturation, the water absorption rate of loess gradually decreases as the number of reinforcement cycles increases. When the number of reinforcement cycles is the same, the higher the grouting saturation of the sample and the lower the water absorption rate. This is because a higher grouting saturation results in a higher content of calcium carbonate generated, reaching a maximum of about 19%, and a lower porosity. Consequently, the water absorption rate of the loess sample gradually decreases with the increase in the mass ratio of calcium carbonate.

The van Genuchten model is the most widely used mathematical model for fitting soil and water characteristic curves at present, and its specific expression is as follows:

(24) $$\frac{S_{r}-S_{r0}}{S_{r1}-S_{r0}}=\frac{1}{{\left[1+{\left(\alpha s\right)}^{n}\right]}^{m}}$$

**Figure 4:**
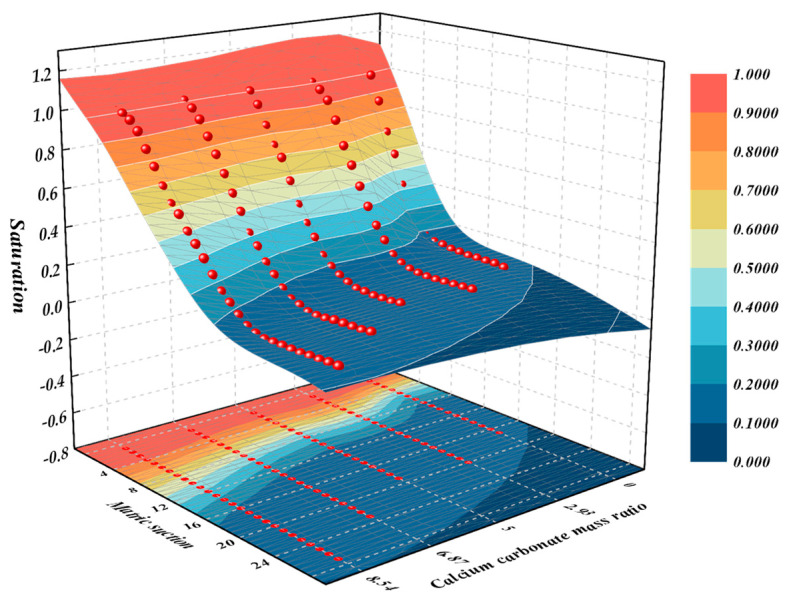
Soil-water characteristic curves of solidified loess under different mass ratios of calcium carbonate.

As shown in Figure 4, MICP has a significant effect on SWCC. Treated loess (Ca/C mass ratio greater than 0) has a higher intake value and faster dehumidification rate than untreated loess (Ca/C mass ratio 0), while there is little difference in the saturation corresponding to the residual state between them. When the matric suction was greater than the intake value, the SWCC of MICP-treated loess was always higher than that of untreated loess under the same matric suction condition. With the increase in MICP treatment times, the mass ratio of calcium carbonate gradually increased, and SWCC was always higher than the curve after the previous MICP treatment in the first four MICP treatments. By the fifth MICP treatment, SWCC was almost the same as the fourth MICP treatment, indicating that SWCC did not change after the mass ratio of calcium carbonate reached 0.0854.

## 3. Experimental Design of MICP Technology and Plant Straw Solidified Loess

### 3.1. Test Material

The loess used in the test was taken from Jingyangkou Town, Shaanxi Province, and the loess sample belongs to Malan loess of the Late Pleistocene (Q3) Quaternary. Its basic physical parameters are shown in Table 1. According to the standard of the 2019 soil test method, after drying the soil sample, large pieces of gravel and sand were screened by 2 mm such that the particle size of the loess soil sample was less than 2 mm. Soil samples were subjected to shear and unconfined compressive tests. Bacteria are usually most abundant in the topsoil (0–10 cm), because the topsoil is usually rich in organic matter and nutrients suitable for bacterial growth and reproduction. The soil sampled in this study was surface soil ranging from 0 to 10 cm, and its mineral composition was mainly quartz, clay minerals, and carbonate minerals. In this paper, the water retention ability of loess in the natural state, moderate water content is conducive to the survival and activity of microorganisms, and provides a good environment for MICP process. The pore structure of loess is relatively small, and the thickness of the solidified layer formed during solidification is limited, mainly concentrated in the surface layer. This property makes the application of MICP technology in loess solidification challenging, but also provides an opportunity for further research to explore how to enhance the deep solidification effect by improving the technology.

The experimental instruments and methods used in this study are as follows.

Constant Temperature Shaking Incubator (Suzhou Jiemei Electronics Co., Ltd., Suzhou, China): This is used to cultivate *Bacillus pasteurii* under controlled temperature and shaking conditions to promote its growth and the production of urease. Conductivity Meter (Shanghai Leici, Shanghai, China): This measures the changes in conductivity of the solution during the urea hydrolysis reaction, allowing for the calculation of urease activity. The change in conductivity is directly proportional to the amount of urea hydrolyzed. Direct Shear Test Equipment (Hebei Better United Test Equipment Co., Ltd., Cangzhou, China): This equipment is used to measure the shear strength of loess. By applying shear force, it measures the shear strength parameters (cohesion and internal friction angle) of soil samples under different conditions. Unconfined Compressive Strength Test Equipment (Xianxian Rushi Technology Co., Ltd., Cangzhou, China): This is used to determine the unconfined compressive strength of soil samples. Axial pressure is applied, and the failure strength of the samples is recorded. Scanning Electron Microscopy (SEM) (Hitachi, Japan): This is used to observe the microstructure of soil samples treated with microbially induced carbonate precipitation (MICP). SEM can magnify the surface of soil samples, clearly showing the distribution of calcium carbonate crystals and the arrangement of soil particles. Mercury Injection Porosimetry (MIP): This is used to measure the pore distribution characteristics and permeability of soil samples. Through MIP testing, parameters such as total pore volume, average pore size, and porosity of the soil samples can be obtained, allowing for an analysis of the changes before and after MICP treatment.

The experimental bacterium was *Bacillus pasteurelli* (US Strain Preservation Center No.: DSM = ATCC 11859). The preparation and culture of the bacterial solution and medium were as follows. The culture medium was cultured with CASO AGAR+ urea (20 g/L) at 30 °C for 2 days with constant temperature and oxygen. Quantities of 5 g NaCl, 20 g AGAR, and 900 mL sterile water were added, mixed evenly, and the PH was adjusted to 7.3 with 1 mol/L NaOH. This was sterilized at 121 °C for 15 min, and 20% urea as added to 100 mL solution when the temperature dropped to 60 °C, shaken well, and set aside. A certain quantity of bacteria was selected from the inclined surface of the test tube on the aseptic operating table using the inoculation ring and directly inoculated into the liquid medium, which was finally moved to an electric constant temperature oscillation tank. The oscillation rate was adjusted to 150 rpm, and the liquid was cultured at 30 °C until the turbidity appeared. The bacterial optical density (OD_600_) measured by spectrophotometry was 0.8.

The cementing fluid was 1 mol/L urea solution and 1 mol/L calcium chloride solution in a 1:1 mixture. Wheat and flax straw were taken from Jingyuan, Gansu Province and cut into 10 mm segments. Wheat and flax are the main cash crops in the region: wheat is mainly used to make flour, flax is commonly used to press oil, and straw, a by-product of agriculture made of feed and fuel, is mostly burned. The characteristics of strong toughness, high strength, and large yield provide support for its application in improving the bearing capacity of foundation, strengthening soil, and slope protection. The ryegrass seed used in the experiment was the domestic Yunseye Dongmu 70 seed. Because of its high survival rate and adaptability to soil and climate in the loess area, it has become a common grass seed used in the study of plant growth adaptability.

### 3.2. Test Scheme

This experiment studied the shear strength of solidified loess by MICP technology combined with wheat and flax straw. The direct shear test was set near the optimal water content (16%), and soil sample densities of 1.7 g/cm^3^ and 1.8 g/cm^3^ were obtained by mixing a static pressing method with 5 experimental groups, as follows: aseptic water group (no curing material was added, that is, blank control), cementing fluid group (only aseptic cementing fluid was added to prove that cementing fluid had no effect on loess strength), bacterial fluid group (MICP technology curing), bacterial fluid + wheat straw group (combined curing 1) and bacterial fluid + flax straw group (combined curing 2).

This experiment aimed to investigate the unconfined compressive strength of solidified loess reinforced with MICP technology and wheat straw. During the unconfined compressive strength testing, soil samples with densities of 1.7 g/cm^3^ and 1.8 g/cm^3^ were prepared using the static pressing method. Five experimental groups were established, following the same setup as in the direct shear test. Additionally, the experiment was designed to explore the alterations in the microscopic properties of loess reinforced through MICP technology. Therefore, two experimental groups were set up, namely, the loess soil samples treated with MICP technology and the untreated loess soil samples, and two samples in each group were prepared by mixing static pressing method with the same sample density of 1.8 g/cm^3^.

This experiment was intended to study whether the loess treated with MICP technology met the plant growth conditions or affected the plant growth. Therefore, the soil samples directly treated with MICP (mixing method) were carried out in the test soil tank, a blank control was set up (without adding any treatment reagents), and the growth process was recorded by culture at room temperature.

Preparation of bacterial solution: Take out a certain amount of cementing solution in the beaker, add an appropriate amount of cultured Bacillus pasteuris solution, prepare the bacterial solution with 10% mass fraction, add 1 mol/L ammonium chloride solution, adjust the PH value of the bacterial solution to 8, and refrigerate for use.

Preparation of soil sample: The screened and dried loess was taken out and put into the dryer for drying and cooling. The soil sample was prepared by hydrostatic pressure with water spraying mixed with soil according to the set target moisture content (16%) and the design density.

Test soil samples were made according to the test plan, and the solution and dry soil were mixed within 10 min (the soil sample with plant straw added was added according to 1% mass fraction), then added into the mold, and static pressure was carried out by jack.

The prepared soil sample was put into a fresh-keeping bag and cultured in a constant temperature and humidity box at 25 °C with 95% relative humidity for 3 days. Soil samples for ryegrass growth adaptability test were cultured at room temperature for 3 days. The soil samples used for SEM scanning electron microscopy test and mercury injection test (MIP) were cultured and cut into 10 mm × 10 mm × 10 mm squares, and then dried in the oven (105 °C, 24 h) for later use. The quantity of carbonate in loess samples was analyzed by SEM technology, and the chemical elements of the samples were analyzed by XRF tests. The relationship between the microstructure and chemical composition of loess was better understood by combining SEM and XRF test.

### 3.3. Factors Affecting Bacterial Growth

In this study, the turbidimetric method and Cary100 ultraviolet spectrophotometer of Agilent Company (Santa Clara, CA, USA) were used to measure the absorbance value (OD_600_) of Sarcina batcheri bacterial liquid at 600 nm wavelength. The conversion formula between bacterial concentration and OD_600_ value is as follows:

(25) $$Y=8.59\times 10^{7}\times Z^{1.3627}$$

During the growth and reproduction of bacteria, urease is continuously produced and accumulated, and the activity of urease in bacterial fluid changes accordingly. Urease activity is defined as the rate of the urea hydrolysis reaction catalyzed by the enzyme under specific conditions. A faster catalytic conversion rate indicates a higher level of urease activity. Various methods exist for determining urease activity, with the conductivity method proposed by Whiffin being the most widely used. The principle behind this method is that the urea hydrolysis reaction alters the number of anions and cations in the solution, resulting in a change in the solution’s conductivity. Specifically, a conductivity change of 1 millisiemens per minute (mS/min) corresponds to an urea hydrolysis amount of 11.11 millimoles (mmol). By measuring the change in electrical conductivity over a given period, one can calculate the range of urease activity. The specific calculation formula for this is as follows:

(26) A=ΔE/t×11.11×m

**Figure 5:**
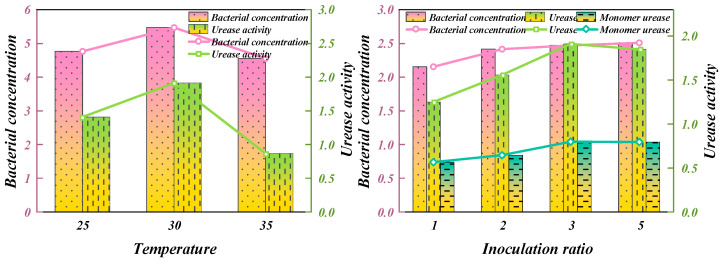
Effect of temperature on bacterial concentration and urease activity.

Temperature has an important effect on the growth of bacteria. Appropriately increasing temperature is conducive to bacterial metabolism, promoting its growth and improving urease activity, but too high a temperature will lead to the degeneration and inactivation of urease, which is essentially protein. In an appropriate temperature range, the concentration and activity of bacteria can be optimized. Under the condition of 1% inoculation ratio, the same batch of culture medium inoculated with Bacillus pasteurella was continuously cultured in a constant temperature oscillation chamber for 30 h (rotating speed of 121 rpm), the temperature was set to 25 °C, 30 °C, and 35 °C, respectively, and then the OD_600_ and urease activities were detected. The test results are shown in Figure 5.

As illustrated in Figure 5, the temperature range of 25–35 °C exerts a notable impact on both the concentration and urease activity of *Bacillus pasteurii*. As the temperature rises, the bacterial concentration gradually increases, peaking at 30 °C. Beyond this point, as the temperature continues to climb, the bacterial concentration begins to decrease. The change in urease activity was basically consistent with the concentration of bacteria, indicating that 30 °C was the best temperature for the expanded culture of bacteria. In the subsequent expanded culture of Bacillus pasteurelli, the culture temperature was set to 30 °C. *Bacillus pasteurelli* showed strong adaptability under changing field conditions and was able to maintain certain survival ability under temperature fluctuations and humidity changes. Its spore-forming capacity and metabolic regulatory mechanisms enabled it to survive adverse environmental conditions and regain activity when conditions improve.

In the process of expanded culture, the inoculation ratio also had a certain influence on the growth rate and activity of the bacteria. The inoculation ratios of 1%, 2%, 3%, and 5% were designed in the experiment, and the bacteria were cultured at the same temperature and pH. The growth of the bacteria was reflected by its concentration (OD_600_ value), urease activity, and monomer urease activity, as shown in Table 2.

As demonstrated in Table 2 and Figure 5, a higher inoculation ratio facilitates achieving the desired bacterial solution concentration within a shorter timeframe, whereas a lower ratio typically necessitates a longer duration to attain the corresponding concentration. Furthermore, the higher inoculation ratio exhibits superior enzyme activity and monomer enzyme activity compared to the lower one. The findings indicate that by increasing the inoculation ratio of Bacillus pasteurella during expanded culture, the urease activity of its monomer can be mitigated. However, bacterial growth is often rapid, and in many instances, overnight culture suffices to attain a high concentration. Consequently, a general vaccination ratio of 1% is usually sufficient to meet the experimental bacteria requirements.

**Figure 6:**
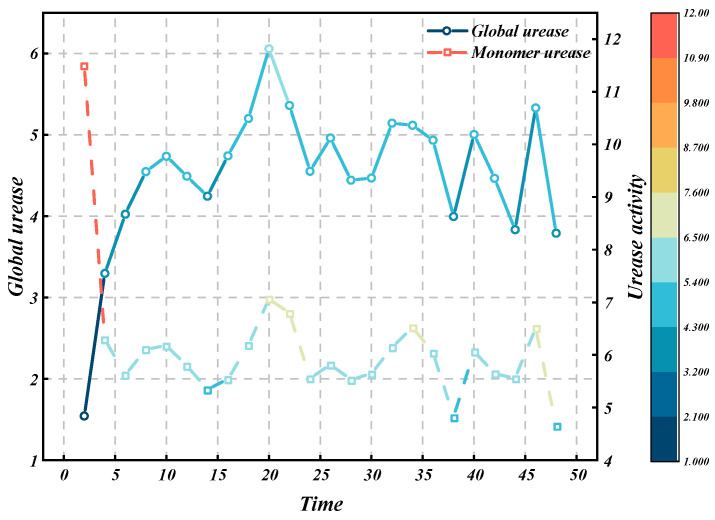
Urease activity curve with time.

While measuring the amount of bacteria, the corresponding urease activity was monitored in real time. The pipette was used to absorb 2 mL of the bacterial liquid to be measured and mixed with 18 m urea solution with a concentration of 1.5 mol (10-fold dilution), and the urea hydrolysis reaction began. The conductivity probe was immediately inserted into the liquid to be measured for a period of time to measure the change in the conductivity of the mixed solution, and then the obtained data were substituted into Equation (26) to calculate the enzyme activity value. Each time point corresponds to the urease activity measured at that point, and the relationship between the two is obtained, as shown in Figure 6.

Compared with Figure 6, it can be seen that the change trend of urease activity in the fluid of Sarcina pasteuri was basically the same as its growth trend. In the logarithmic period of bacteria, the total urease activity of *Pasteuria* spp. liquid increased with the increase in culture period, and the total urease activity of *Pasteuria* spp. liquid reached a peak of 6.1115 mmol after 20 h. The change trend of urease activity of unit bacteria was roughly opposite to that of the overall urease activity. When the culture time exceeded 6 h, the urease activity of unit bacteria first reached a minimum value of 5.6048 mmol and then began to fluctuate, indicating that the urease production efficiency of Sarcina Pasteuri reached the limit at this time. Since the overall urease activity of the bacterial solution is more important in MICP testing, considering the economy and suitability of the test, this study selected the third-generation Pasteuria spore Sarcina solution cultured at 30 °C and 120 rpm for 20 h to carry out subsequent experimental research.

## 4. Test Results and Analysis

### 4.1. Soil pH and Eh and Electron Microscope Scanning

**Figure 7:**
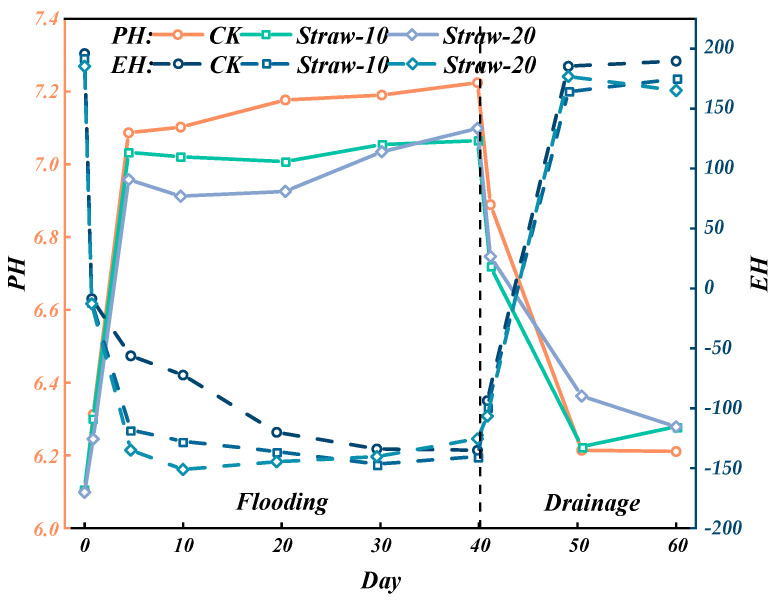
Changes in soil *pH* and *Eh*.

Figure 7 illustrates the variations in soil *pH* and Eh during the watering and drying cycles. Throughout the entire “watering–drying” phase, significant changes were observed in both soil *pH* and *Eh*. Following soil flooding, the *pH* gradually increased towards neutrality. Upon soil drying, the *pH* reverted to levels close to those prior to flooding. Notably, the incorporation of straw mitigated the rise in soil *pH* during the flooding stage, potentially due to straw decomposition in the soil. Within the first day of flooding, the *Eh* of all three treatments rapidly declined below 0 mV. Conversely, after soil drainage, *Eh* swiftly rose above 160 mV. In this study, straw application notably exacerbated the initial 20-day decline in soil *Eh*, which subsequently stabilized at approximately −140 mV. After 20 days of flooding, no significant impact of straw application on soil *Eh* or *pH* was observed during the drying phase.

**Figure 8:**
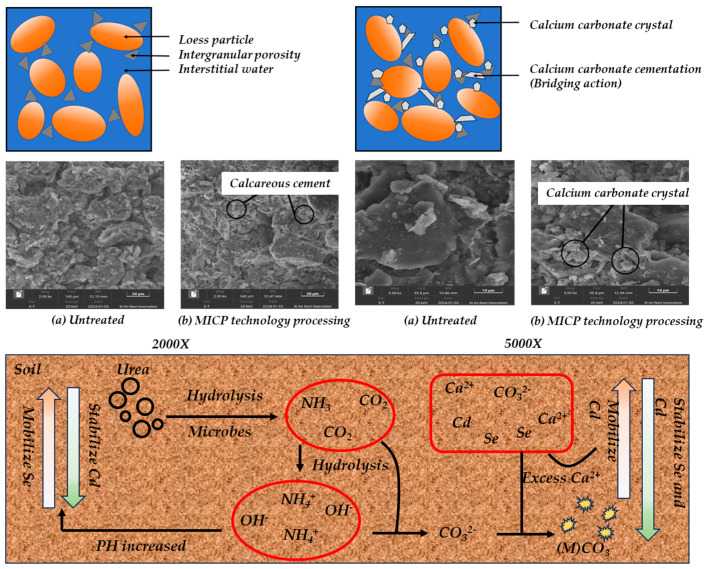
SEM and microscopic process.

Through scanning electron microscopy, the microstructure of soil sample particles before and after MICP treatment can be seen (magnified to 2000 times—see Figure 8). The untreated soil samples showed large pores, relatively loose particle arrangement, and no obvious calcareous cementation on the surface. Following MICP treatment, the soil sample pores were visibly filled with tightly packed calcium carbonate particles, resulting in a significant enhancement in calcium cementation. Upon further examination at 5000× magnification, it became clear that calcium carbonate crystals had formed between the soil particles after MICP application. In contrast, the untreated soil particles exhibited no obvious calcium carbonate crystals on their surfaces. Instead, the white adherent substance observed could be attributed to the original organic matter or inorganic salt particles present in the soil sample.

The application of MICP technology had a profound impact on pore filling between soil particles, resulting in substantial calcium cementation. This process transformed the connection mode between soil particles from “point contact” to “surface contact”, achieving cementation and a “bridging effect” that significantly enhanced the overall strength of the sample. The impact on pore filling is illustrated in Figure 8. MICP technology can produce a large amount of calcium cementation between soil particles in a short time, and it can be concluded that MICP technology can significantly improve the degree of cementation between loess particles. This is consistent with the microscopic characteristics of soil particles observed by Liu Jianxing et al. [35] when studying the microscopic properties of silt solidified by MICP technology, which further verifies the cementation and “bridging” effect of MICP technology on loess soil particles and proves the improvement effect of MICP technology on loess strength from a microscopic perspective.

### 4.2. Analysis of Shear Strength of Loess

**Figure 9:**
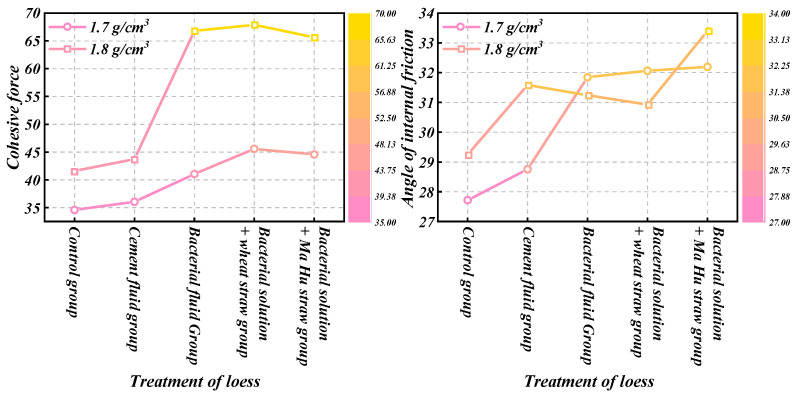
Cohesion c, internal friction, and angle φ of loess under different treatment methods.

Based on the results of the direct shear tests, we obtained the shear strength envelope and its parameters, including cohesion (c) and internal friction angle (φ). By compiling the shear strength parameters of loess across various densities and treatment methods, we generated a comparative diagram, depicted in Figure 9. The diagram reveals that loess treated with microbially induced calcification (MICP) technology exhibitedenhanced cohesion and internal friction angle. Specifically, at densities of 1.7 g/cm^3^ and 1.8 g/cm^3^, the cohesion of the experimental groups with bacterial solution was approximately 30% and 50% higher, respectively, compared to those without bacterial solution. Additionally, the internal friction angles were approximately 15% and 5% higher, respectively. These findings suggest that MICP technology significantly improves both the cohesion and internal friction angle of loess, thereby notably enhancing its shear strength. The cementing fluid set in the experimental group excluded the possible influence of the cementing fluid in the bacterial solution on the strength of loess. When the density was 1.7 g/cm^3^, the loess cohesion of MICP technology combined with plant straw curing treatment increased again, but when the density was 1.8 g/cm^3^, the loess cohesion of MICP technology combined with plant straw curing treatment did not increase much, indicating that at the low density of 1.7 g/cm^3^, the loess cohesion increased by MICP technology combined with plant straw curing treatment. The addition of plant straw played a better role in solidifying loess.

Through a thorough analysis of the test results, coupled with the understanding of the action mechanism of MICP technology, it becomes evident that *Bacillus pasteurii* can induce the precipitation of calcium carbonate, leading to the formation of calcium carbonate crystals. These crystals effectively cement and fill the gaps between loess particles, thereby significantly boosting the cohesion of the loess. However, the impact of calcium carbonate crystallization on soil compactness and surface roughness is relatively minor, resulting in a negligible effect on the internal friction angle of the loess. Furthermore, straw possesses inherent tensile strength and extensibility, making it a suitable reinforcement material. The fiber structure of plant straw can form physical support between soil particles and enhance the overall structural stability of soil. The long fibers of straw can form a network structure in the soil and increase the compressive and tensile resistance of the soil. In addition to utilizing MICP technology, incorporating plant straw into reshaped loess of low density can markedly enhance its shear strength and deformation resistance. Given that there is a scarcity of research on the use of plant straw as a reinforcement material in slope protection, particularly in the context of applying MICP technology for loess reinforcement, further investigation in this area is crucial. Such research will facilitate a deeper understanding of the effects and mechanisms of the combined improvement technology involving plant straw reinforcement and MICP technology, ultimately paving the way for its application in strengthening loess.

### 4.3. Unconfined Compressive Strength Analysis

**Figure 10:**
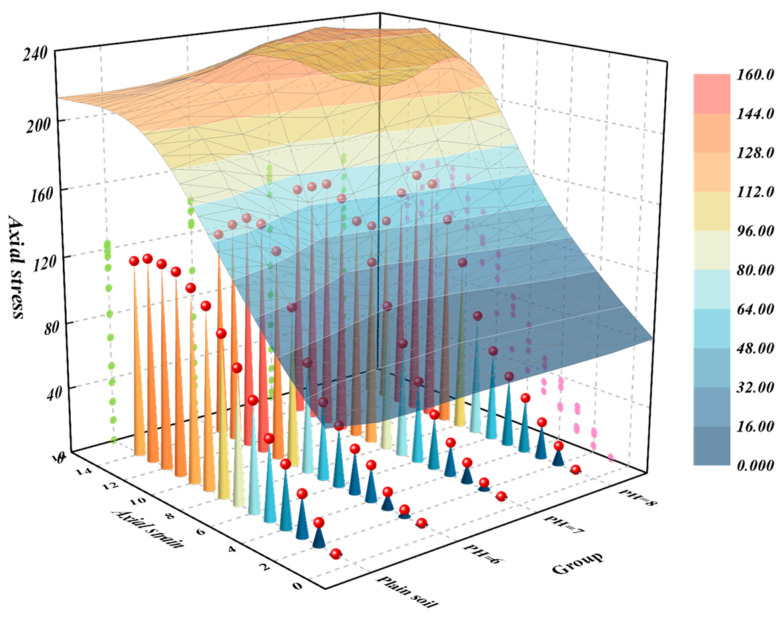
Influence curve of PH of cementing fluid on unconfined compressive strength.

The results of the unconfined compressive strength (UCS) tests are presented in Figure 10. Upon examination, it is evident that as the pH value of the cementing fluid increases, the failure resistance of the samples also continues to rise. Specifically, the axial stress attains its peak when the pH value of the cementing fluid reaches 8. Additionally, in comparison to plain soil, the UCS of soil strengthened by microorganisms exhibits an increase of 15.2%, 22.5%, and 24.7% when the pH value of the cementing fluid is set to 6, 7, and 8, respectively. This demonstrates that treating the soil with bacterial solutions containing cementing fluids of different pH values can enhance its UCS. Moreover, the strength of the soil samples is maximized when the pH of the cementing solution is adjusted to 8.

**Figure 11:**
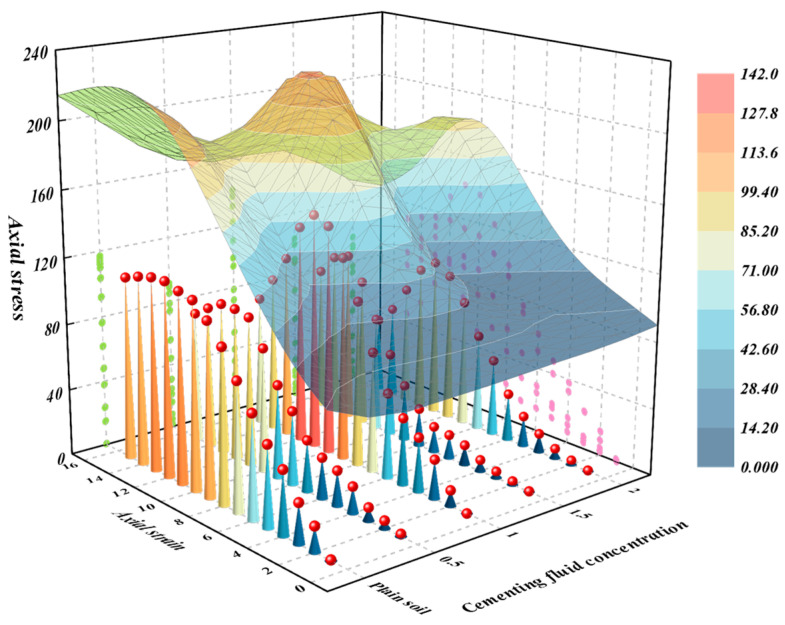
Influence curve of cementing fluid concentration on unconfined compressive strength.

The results of the unconfined compressive strength tests are depicted in Figure 11. Upon analysis, it is apparent that as the concentration of the cementing fluid increases, the failure resistance of the sample initially rises, but subsequently declines. When the concentration of cementing fluid is 1.0 mol, the axial stress reaches the maximum UCS. In comparison to plain soil, the UCS of the soil sample only increases by 24.7% when the cementing fluid concentration is 1.0 M. Conversely, when the cementing fluid concentration is set to 0.5 M, 1.5 M, or 2.0 M, the UCS of the samples is lower than that of plain soil. This indicates that there exists a critical concentration for the cementing fluid. Specifically, the UCS of the soil is enhanced only when the cementing fluid concentration is precisely 1.0 M.

**Figure 12:**
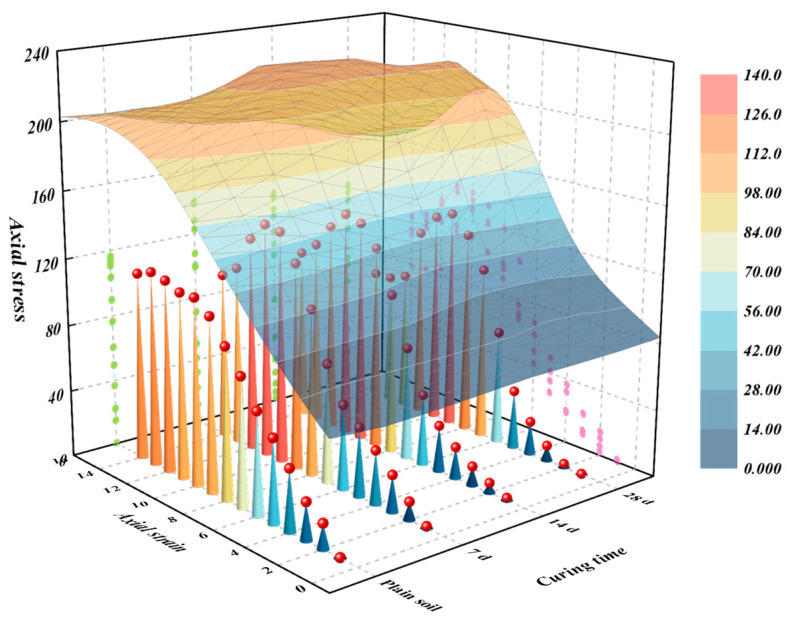
Influence curve of curing time on unconfined compressive strength.

The results of UCS tests are presented in Figure 12. Upon analysis, it is evident that as the curing time increases, the failure resistance of the sample exhibits a trend of initial decline followed by an increase. Specifically, the UCS attains its peak value when the soil sample is cured for 7 days. On the other hand, compared with plain soil, the UCS of the soil strengthened by microorganisms increased by 24.7%, 20.2%, and 23.6% when the curing time was 7, 14, and 28 days, respectively. Subjecting soil samples to microbial treatment and a specified curing period can enhance their UCS. Notably, a curing time of 7 days yields the most significant increase in UCS. However, on average, the improvement in UCS remains relatively consistent across different curing times.

### 4.4. Pore Distribution Characteristics and Permeability Analysis

**Figure 13:**
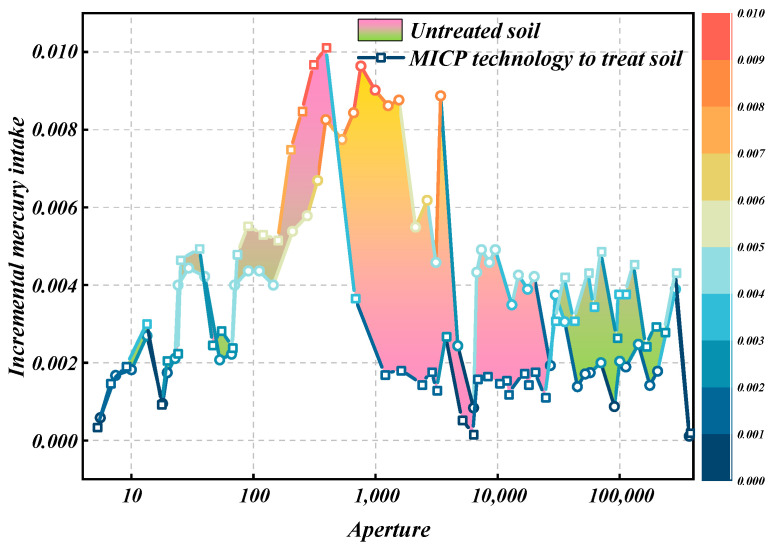
Incremental intrusion vs. pore size.

Utilizing the mercury injection test (MIP), the pore distribution characteristics and permeability indices of loess, both pre- and post-MICP treatment, were determined, and are presented in Table 3. Furthermore, based on the MIP findings, the relationship curve depicting the variation in sample aperture with respect to the mercury injection increment, both before and after MICP treatment, is plotted in Figure 13.

The data in Table 3 reveal that loess treated with MICP technology exhibits decreases in total pore volume, total pore area, average pore size, permeability, and porosity, while the tortuosity increases. This analysis indicates that MICP technology effectively fills the pores between loess particles with calcium carbonate crystals. As a result, the pores are occupied by a substantial number of these crystals, leading to a reduction in total pore volume and average pore diameter, while the total pore area paradoxically increases (potentially due to complex pore geometries formed by the crystal deposition). Consequently, the flow path of mercury liquid through the soil pores becomes more tortuous, resulting in an increase in tortuosity.

The circle lines represent the untreated soil, and the square lines represent the pore diameter - mercury intake increment after improvement using MICP technology. It can be seen in Figure 13 that the pore diameter–mercury injection increment curve before and after MICP treatment is very different. According to the size classification of loess pores based on mercury injection test in China, the content of mesopores (pore radius 0.016–0.004 mm) and small pores (pore radius 0.004–0.001 mm) in loess that has not been treated by MICP technology is higher. The content of large pores (pore radius > 0.016 mm) and small pores (pore radius < 0.001 mm) in the treated loess had little change or slightly increased compared with that of untreated loess, while the content of medium and small pores significantly decreased. In general, the loess treated by MICP technology is mainly concentrated on the reduction of middle and small holes, so it can be concluded that the middle and small holes of the loess treated by MICP technology are filled with a large number of calcium carbonate crystals, and the treatment effect is obvious.

Most of the studies were carried out under laboratory conditions, with a lack of field test validation for practical engineering applications. This may cause the feasibility and reliability of the experimental results in practical applications to be questioned. In addition, only Bacillus pasteuris was used as the microbial curing agent in the study, and the possibility of other microorganisms and their influence on the curing effect were not considered, which limits the universality of the study.

## 5. Conclusions

In this paper, the strength characteristics and microscopic mechanism of the loess solidified by MICP technology and plant straw were studied, and the permeability conditions of loess under MICP technology were obtained. The experimental materials included loess, Bacillus pasteurella, specific cementing fluid, and plant stalks from specific areas. Various experimental groups were set up to study the shear strength, unconfined compressive strength, microscopic properties, and plant growth adaptability of loess to explore the influencing factors of bacterial growth. Specific conclusions are as follows.

1. The pores of the soil sample treated by MICP technology are filled with obvious calcium carbonate particles, the particles are arranged more closely, and the degree of calcium cementation is significantly improved. Magnification to 5000 times observation, after MICP technology treatment, calcium carbonate crystals between soil particles can be clearly observed, while there is no obvious calcium carbonate crystal on the surface of untreated soil particles, and the white adhesion substance on the surface may be the original organic matter or inorganic salt particles in the soil sample.
2. When the density is 1.7 g/cm^3^, the loess cohesion of MICP technology combined with plant straw curing treatment increases again, while when the density is 1.8 g/cm^3^, the loess cohesion of MICP technology combined with plant straw curing treatment does not increase much. At the low density of 1.7 g/cm^3^, the loess cohesion of MICP technology combined with plant straw curing treatment does not increase much. The addition of plant straw plays a better role in solidifying loess.
3. When the concentration of cementing fluid reaches 1.0 M, the precipitation mode of CaCO_3_ will change, and the newly generated calcium carbonate will preferentially precipitate on the existing body instead of forming crystal nuclei at new points. Larger calcium carbonate crystals will be formed and deposited between silt particles and clay particles, enhancing the structural stability and strength of the soil mass.
4. The loess that has not been treated by MICP technology has more mesopores (pore radius 0.016–0.004 mm) and small pores (pore radius 0.004–0.001 mm). The content of large pores (pore radius > 0.016 mm) and small pores (pore radius < 0.001 mm) in the treated loess had little change or slightly increased compared with that of untreated loess, while the content of medium and small pores significantly decreased.

In practical engineering, the physical and chemical properties of soil can be very different from laboratory conditions, and it is difficult to control some of the outdoor influencing factors, such as soil type, moisture content, temperature, and pH. These changes may affect the effectiveness of the MICP process and the activity of the bacteria. Rainfall can lead to soil saturation, affect its pore water pressure and effective stress, and thus affect the shear strength and stability of the soil. Future research should pay more attention to long-term durability testing, especially in practical engineering environments, to ensure the reliability and effectiveness of treated loess in long-term use.

## Figures and Tables

**Table 1:** Physical parameters of loess in the study area.

Density g/cm^3^	Natural Moisture Content %	Maximum Dry Density g/cm^3^	Optimal Moisture Content %	Liquid Limit %	Plastic Limit %
1.87	17.4	1.98	16.2	28.3	18.5

**Table 2:** Components of bacterial medium.

Inoculation Ratio	Culture Time (h)	OD_600_	Urease Activity (ms/cm/min)	Monomer Urease Activity (ms/cm/min)
1%	24	2.136	1.26	0.59
2%	20	2.403	1.62	0.67
3%	18	2.412	1.9	0.79
5%	18	2.42	1.84	0.76

**Table 3:** Mercury injection test results.

Sample Number	Capillary Utilization Rate (%)	Pore Volume (ml/g)	Total Pore Area (m^2^/g)	Mean Aperture (mm)	Porosity (%)	Permeability (mdarcy)	Tortuosity
3-1	51	0.2593	8.240	125.86	37.1	2247.73	4.89
3-2	37	0.2077	8.808	94.31	33.1	460.18	13.22

## Data Availability

The data in this study are available on request from the authors. The data are not publicly available because they are part of ongoing studies.
